# A case of recurrent malignant phyllodes tumor undergoing nipple-sparing mastectomy with immediate breast reconstruction

**DOI:** 10.1186/s40792-020-01022-5

**Published:** 2020-11-25

**Authors:** Emi Morioka, Masakuni Noguchi, Miki Noguchi, Masafumi Inokuchi, Ken-ichi Shimada, Akihiro Shioya, Akane Aikawa, Hiroshi Minato, Mitsuharu Earashi

**Affiliations:** 1 Department of Breast and Endocrine Surgery/Breast Center, Kanazawa Medical University Hospital, 1-1, Daigaku, Uchinada-machi, Kahoku-gun, Ishikawa 920-0293 Japan; 2 Department of Plastic Surgery, Kanazawa Medical University Hospital, 1-1, Daigaku, Uchinada-machi, Kahoku-gun, Ishikawa 920-0293 Japan; 3 Department of Pathology and Laboratory Medicine, Kanazawa Medical University Hospital, 1-1, Daigaku, Uchinada-machi, Kahoku-gun, Ishikawa 920-0293 Japan; 4 Department of Breast Surgery, Toyama Nishi General Hospital, 1019, Shimokutsuwada, Fuchu-machi, Toyama-shi, Toyama 939-2716 Japan

**Keywords:** Malignant phyllodes tumor, Immediate breast reconstruction, Nipple-sparing mastectomy

## Abstract

**Background:**

Although the primary treatment for malignant phyllodes tumor (PT) is complete surgical excision with either breast-conserving surgery or total mastectomy, recent technical advances have led to the adoption of nipple-sparing mastectomy (NSM) with immediate breast reconstruction (IBR).

**Case presentation:**

A 28-year-old woman noticed a mass in her left breast that was rapidly increasing in size. She underwent tumor excision and a histological diagnosis of marked degenerative and necrotic induration suggested benign PT. One year later, however, she was found to have recurrent masses in the left breast on follow-up mammography and sonography. Needle biopsy was performed and the tumor was diagnosed as borderline or malignant PT. She underwent NSM and sentinel lymph-node biopsy with IBR using a tissue expander. Histological examination of the mastectomy specimen showed multiple fibroepithelial tumors with marked stromal overgrowth, focal necrosis, and hemorrhage. Stromal cells showed pleomorphism and a maximal mitotic rate of approximately 25 per 10 high-power fields. The tumor was diagnosed as malignant PT. She did not receive adjuvant chemotherapy or radiation treatment. At 3-year follow-up, the patient remains free of disease and highly satisfied with the cosmetic results.

**Conclusions:**

NSM with IBR is not a contraindication for malignant PT. It is both curative and can offer an appealing cosmetic option for localized malignant PT.

## Background

Phyllodes tumor (PT) is a rare fibroepithelial neoplasm of the breast accounting for less than 1% of all breast tumors [1]. Initially, it was described as cystosarcoma phyllodes [2]. However, the tumors are rarely cystic and the term “sarcoma” tends to overstate the malignant potential. In 1981, the World Health Organization clarified the terminology and adopted the term “phyllodes tumor” [3]. The biological behavior of PTs ranges from relatively benign lesions to highly aggressive malignant lesions. They are pathologically classified as benign, borderline, or malignant based on their histological features, including tumor margins, stromal cellularity, stromal atypia, mitotic rate, and pleomorphism according to the World Health Organization criteria [4, 5]. Malignant PT accounts for 2–45% of all PTs [1].

Surgery is the primary treatment option for malignant PT [6, 7]. However, the extent of surgery remains controversial [8]. Although total mastectomy was considered as standard treatment for patients with malignant PT [5], breast-conserving surgery (BCS) is currently an appropriate treatment option for some patients with malignant PT [9]. Nevertheless, total mastectomy with or without breast reconstruction may be the preferred option in patients with large or recurrent malignant PTs. To date, there have been few reports on the use of implants for immediate breast reconstruction (IBR) after nipple-sparing mastectomy (NSM) for malignant PT [10, 11]. Here, we report a case of recurrent malignant PT undergoing NSM with IBR using a tissue expander/implant, and discuss the validity of IBR after NSM.

## Case presentation

A 28-year old female visited the Toyama-Yatsuo General Hospital in January 2016, because she noticed a mass in her left breast. Mammography and sonography showed a round, well-defined, 5.5-cm mass in the left breast. It was clinically suggested to be a fibroadenoma. She underwent tumor excision through a transverse medial incision in the left breast under general anesthesia. After surgery, a histological diagnosis of marked degenerative and necrotic induration was made suggesting benign PT. In January 2017, however, follow-up mammography showed multiple round or oval tumors with smooth contours (size: 1.2 cm, 1.6 cm, and 1.7 cm in a diameter) in the left breast. Sonography showed four oval or lobulated well-defined masses in the inner quadrant of left breast (size: 2.1 cm, 1.5 cm, 0.8 cm, and 1.2 cm in longest diameter). Ultrasound-guided needle biopsy was performed and the tumor was histologically diagnosed as borderline or malignant PT. Total mastectomy was recommended as the treatment of choice. However, she desired to receive IBR after total mastectomy and visited the Kanazawa Medical University Hospital. Breast magnetic resonance imaging (MRI) showed multiple tumors in the left breast. ^18^F-Fluorodeoxyglucose-positron emission tomography/computed tomography (PET-CT) showed neither distant metastases nor regional metastases. The options for treatment were discussed with the patient. In April 2017, she underwent NSM with IBR using a tissue expander. Sentinel lymph-node (SLN) biopsy was performed using peritumoral injection of radioisotope and subareolar injection of blue dye. After SLN biopsy, the entire breast tissue was subcutaneously removed through a transverse medial incision in the left breast and an axillary incision using wound retractors [12]. Intraoperative histological examination of frozen sections showed that the retronipple biopsy was negative for disease and SLN was not involved. A subpectoral pocket was made by electrocautery and a tissue expander was placed into the pocket to achieve breast symmetry.

**Fig. 1:**
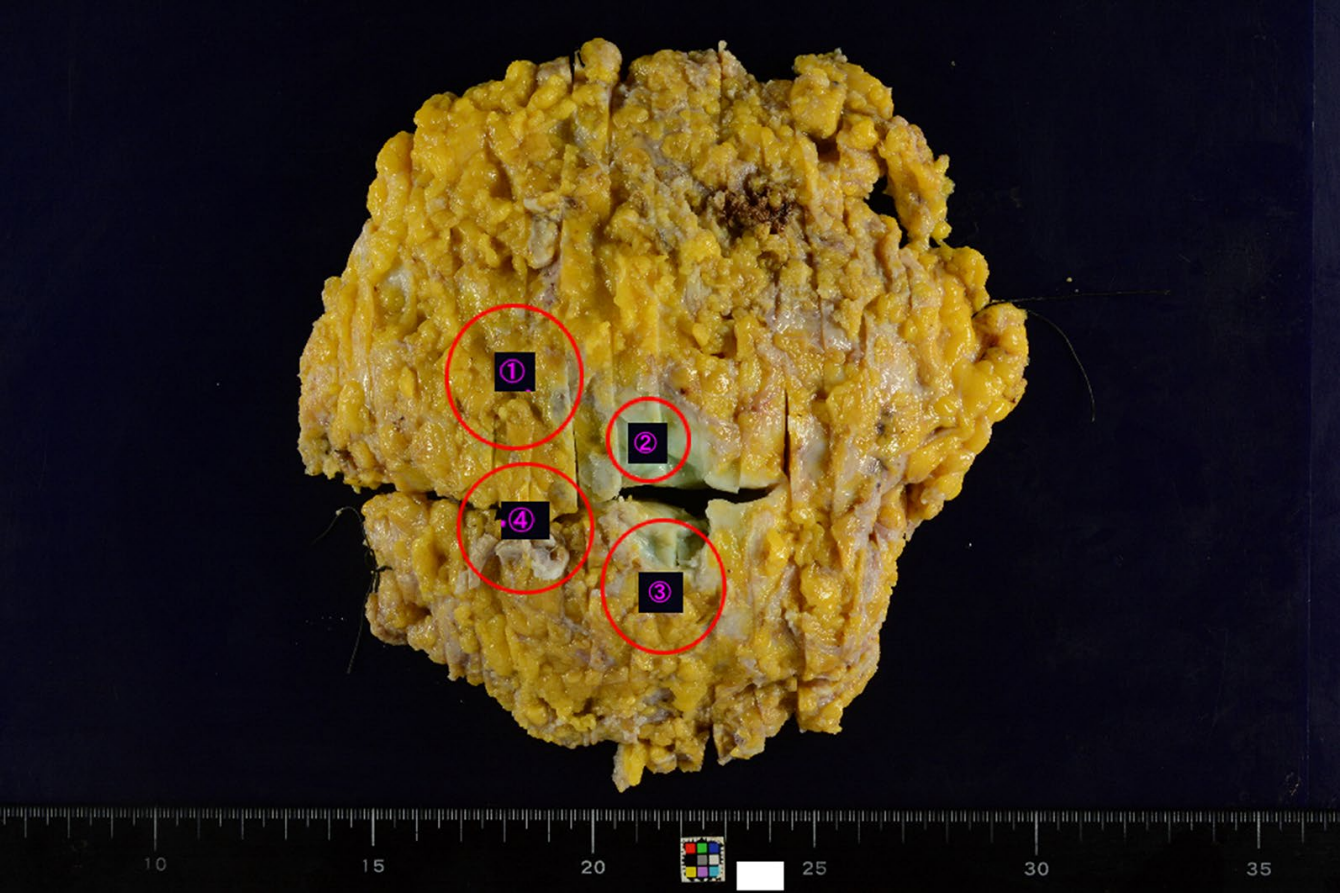
The resected breast tissue and locations of multiple tumors

**Fig. 2:**
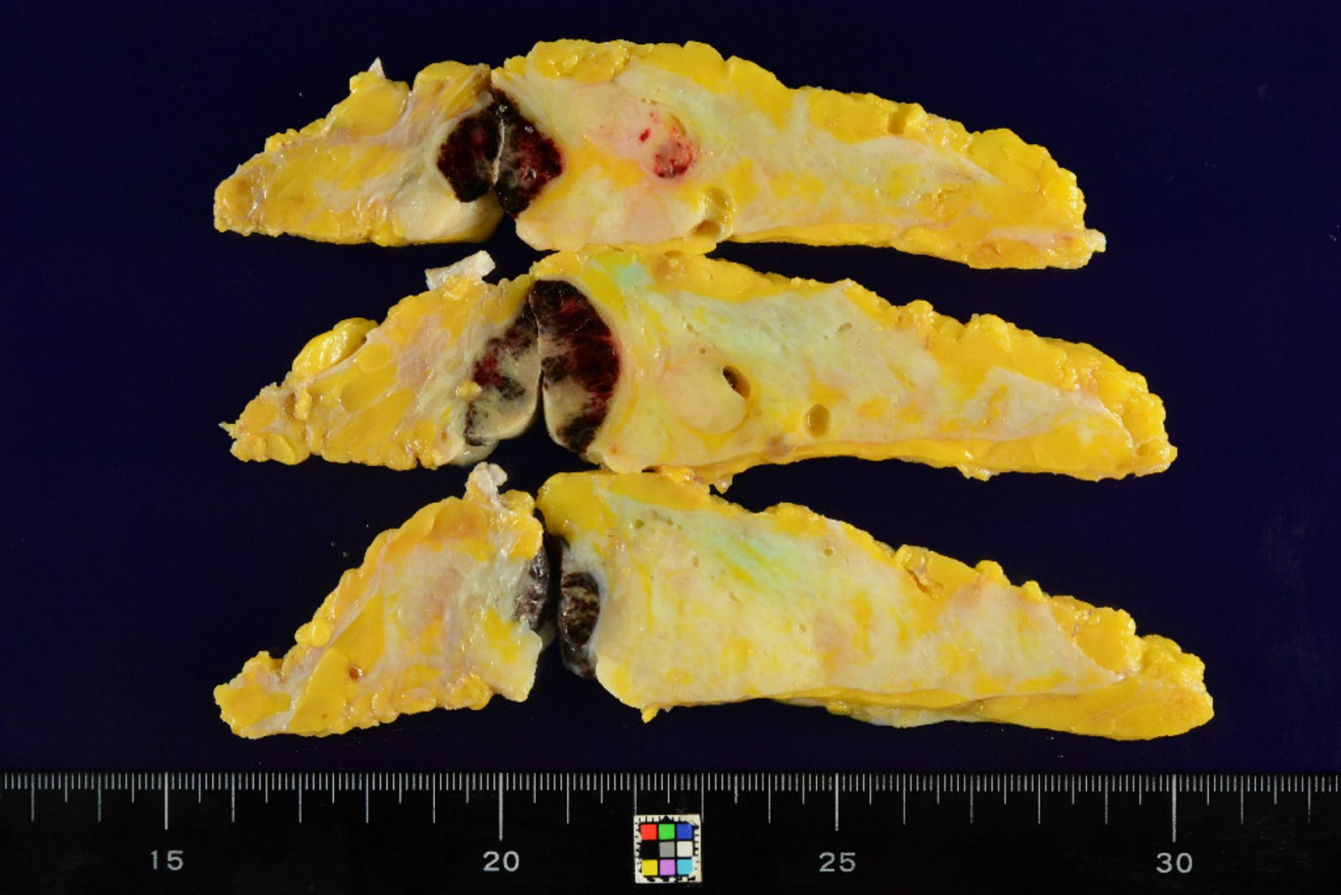
Gross appearance of the cut surface of the resected tumor showed multiple grayish-white, solid, hard masses with hemorrhage and necrosis

**Fig. 3:**
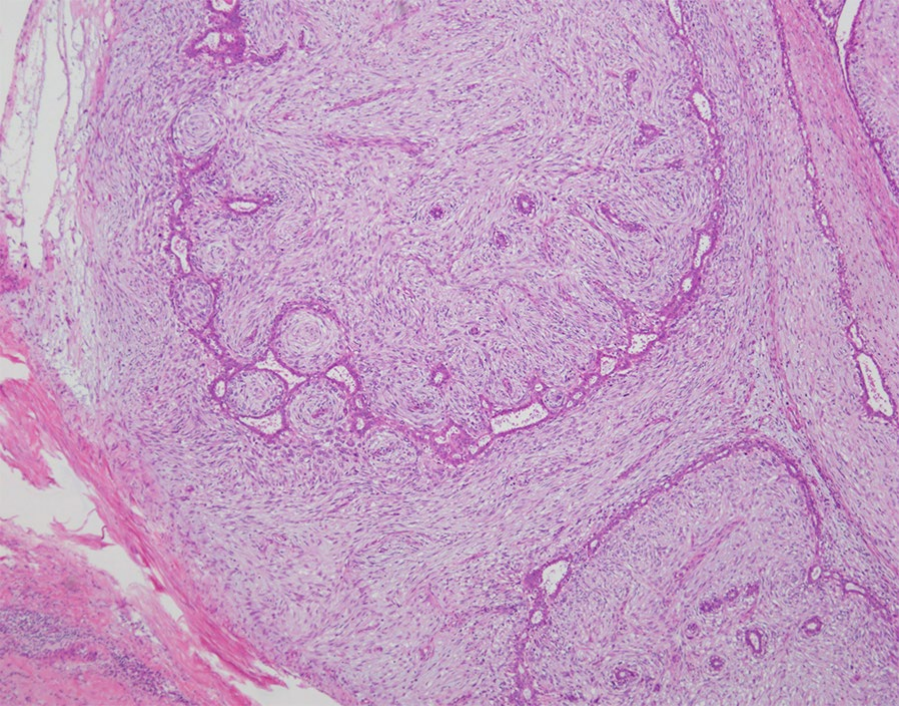
Histological examination of the specimen showed a fibroepithelial tumor with leaf-like growth pattern and stromal condensation (400×) (a tumor in the upper inner quadrant ①)

**Fig. 4:**
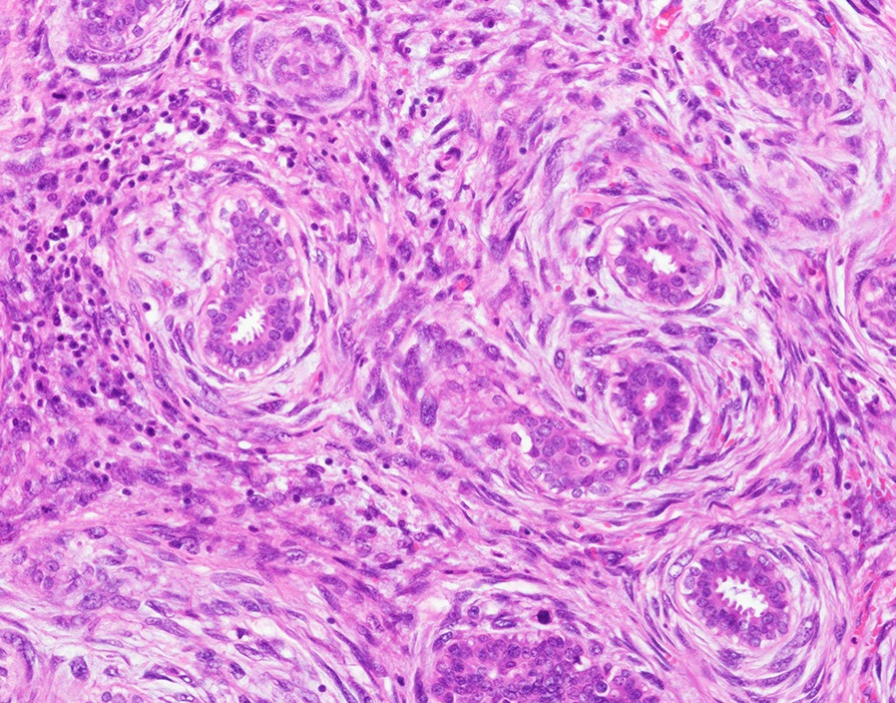
Stroma cells show pleomorphism and > 25 mitoses per 10 high-power fields (200×) (a tumor below the areola ②)

**Fig. 5:**
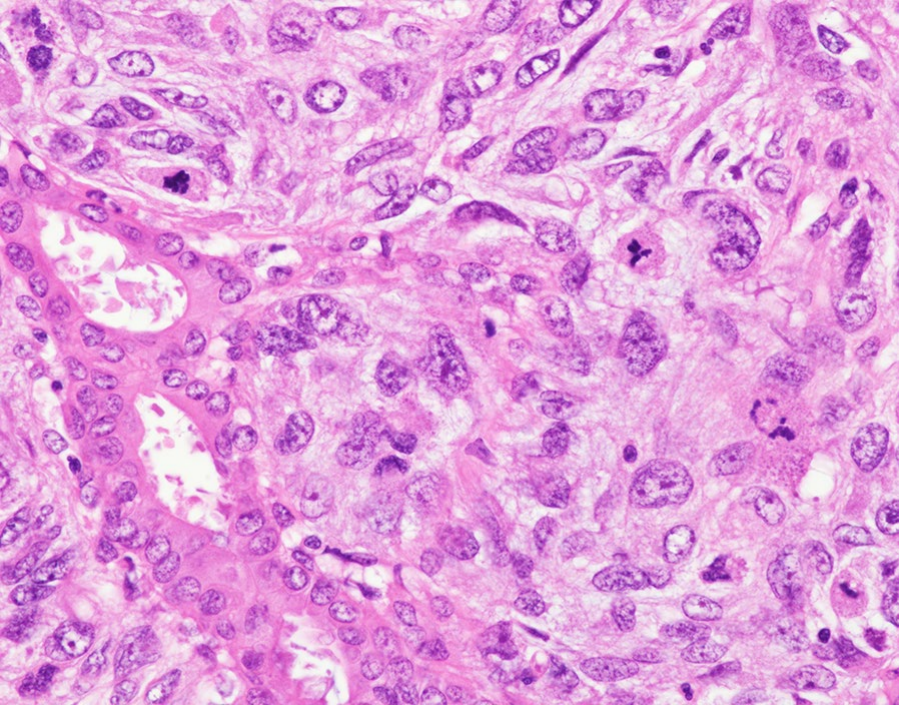
Stroma cells show pleomorphism and > 25 mitoses per 10 high-power fields (400×) (a tumor in the lower quadrants ③)

**Fig. 6:**
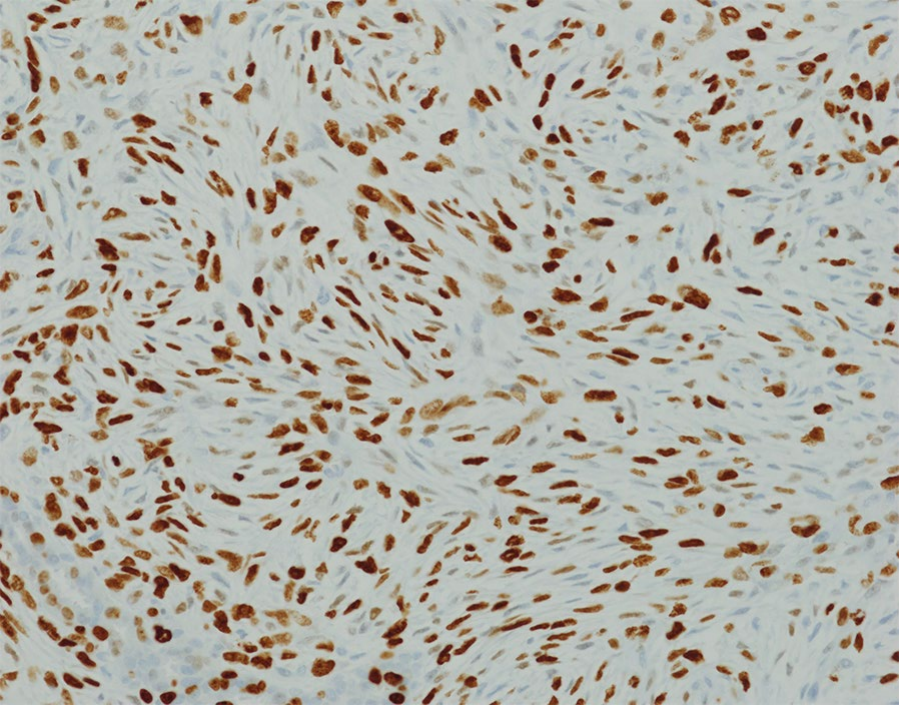
Immunohistochemical staining for Ki67, using the MIB-1 clone (Dako) (400×). Any intensity of nuclear staining indicates a Ki67 positive cell (a tumor in the inner quadrant ④)

**Fig. 7:**
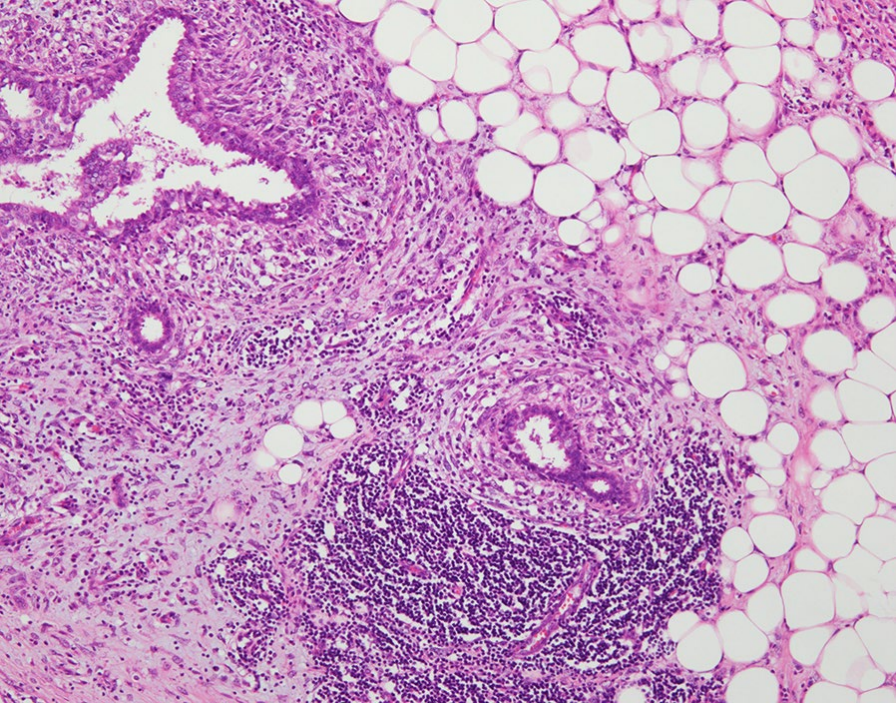
The tumor invaded the surrounded fatty tissue (100×) (a tumor in the inner quadrant ④)

The resected specimen showed multiple tumors, including a tumor 2.3 cm in diameter in the inner quadrant, a tumor 2.3 cm in diameter in the upper inner quadrant, a tumor 0.8 cm in diameter below the areola, and a tumor 3.0 cm in diameter in the lower quadrants (Fig. 1). The gross appearance of the cut surface of the tumor is shown in Fig. 2. Histological examination showed a fibroepithelial tumor with marked stromal overgrowth (Fig. 3), focal necrosis, and hemorrhage. Stromal cells showed pleomorphism with a maximal mitotic rate of approximately 25 per 10 high-power fields (Figs. 4, 5) and maximal Ki-67 proliferation rate of 75% (Fig. 6). The tumor was pathologically diagnosed as malignant PT. Although the tumor infiltrated into the surrounding fatty tissue (Fig. 7), all surgical margins were histologically negative for malignancy.

**Fig. 8:**
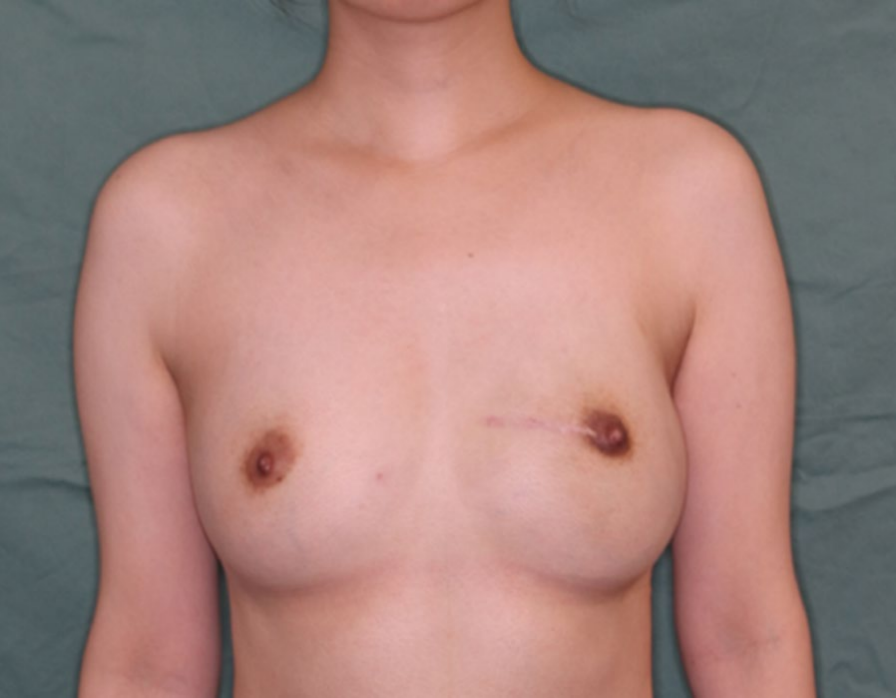
Postoperative view after NSM and breast reconstruction with implant

Postoperatively, expansion of the tissue expander was terminated when it was filled to a volume 20% greater than the volume of the opposite breast. Exchange of the tissue expander for an implant and autologous free fat grafting using the Coleman technique [13] were performed 6 months after surgery. Radiation treatment was not performed for the reconstructed breast. One year after surgery, postoperative breast MRI and PET-CT showed neither local recurrence nor distant metastases and she desired to have a baby. In January 2020, a baby was born without evidence of abnormalities. At 3-year and 4-month follow-up, to date, she remains free of disease and highly satisfied with the cosmetic results (Fig. 8).

## Conclusions

Malignant PT is a rare neoplasm of the breast with an annual incidence of about 2 per million women [14]. Surgery is the primary option for the treatment of malignant PT. Due to its rarity, however, little is known about appropriate surgical management and prognosis after surgical excision of malignant PT. Local recurrence is common in malignant PT, with reported frequencies ranging from 10 to 65% [9]. Radical mastectomy was the treatment of choice in the past [5]. Currently, however, BCS is an appropriate treatment option if negative margins can be achieved with acceptable cosmetic outcome [9]. Nevertheless, total mastectomy should be considered in patients with large or multiple malignant PTs. Radiotherapy has been suggested to decrease local recurrence [7, 15], but no randomized-controlled trials have examined the efficacy of radiotherapy after margin-negative surgery for malignant PT.

Although the primary treatment for malignant PT is complete surgical excision with either BCS or mastectomy, recent technical advances have led to the adoption of mastectomy with IBR. However, there is no consensus on reconstructive options. Reconstructive options for IBR have included submuscular implants and the use of a myocutaneous flap. A few reports described immediate autogenous reconstruction by transverse rectus abdominis musculocutaneous (TRAM) flap and latissimus dorsi (LD) musculocutaneous flap [16–19]. Kobraei et al. [19] reported a case of malignant PT that recurred after mastectomy with immediate TRAM flap reconstruction. The recurrent disease involved the mastectomy bed, TRAM flap, abdominal donor site, and precostal tunnel. They suggested that a delayed reconstruction may be appropriate to confirm a complete resection and to monitor for signs of recurrence. On the other hand, IBR with a tissue expander/implant has advantages over autogenous reconstruction for malignant PT, because these prostheses are inserted submuscularly where they would not impair the detection of local recurrence. Nevertheless, there has been little discussion in the literature regarding IBR with a tissue expander/implant following resection of malignant PT. There have been a few reports on the use of implants for immediate breast reconstruction after NSM for malignant PT (Table 1) [10, 11]. Farias-Eisner et al. [10] reported successful NSM with immediate implant reconstruction with acellular dermal matrix (AlloDerm). Libondi et al. [11] reported successful NSM with immediate implant reconstruction without acellular dermal matrix. After 1 year of follow-up, both of these patients remained free of disease and were highly satisfied with the cosmetic results. Our patient also remains free of disease and is highly satisfied with cosmetic results. When NSM can be performed safely, IBR is an appealing technique for its esthetically pleasing results in patients with malignant PT.

**Table 1:** Clinical profiles of patients with malignant phyllodes tumors undergoing nipple-sparing mastectomy with immediate breast reconstruction using implant

No	Age	Tumor size (cm)	Histology	Surgery	Reconstruction	Cosmetic appearance	Adjuvant therapy	Follow-up (months)	Recurrence	Reference no
1	51	5	MPT	NSM	Implant with AlloMax	Good	None	12	None	[10]
2	19	5.3	MPT	NSM	Implant	Excellent	None	12	None	[11]
3	28	8	MPT	NSM	Tissue expander/implant	Excellent	None	28	None	Present case

Borderline phyllodes tumor was excluded in the study

*MPT* malignant phyllodes tumor, *NSM* nipple-sparing mastectomy

Axillary lymph-node dissection (ALND) is not routinely recommended, since nodal involvement is very rare with less than 1% of patients [15]. The recommended national cancer center network (NCCN) guideline treatment of malignant PT is complete surgical excision without SLN biopsy. Despite the NCCN guidelines recommending against it, however, one in four women underwent axillary nodal sampling in USA [15]. Our patient underwent SLN biopsy, because we concerned about finding an occult breast cancer in the mastectomy specimen rather than the possibility of axillary nodal involvement due to malignant PT. Subareolar injection allows the use of the SLN biopsy technique in patients with multiple tumors [20]. SLN biopsy may be useful to distinguish localized malignant PT without regional disease from malignant PT with regional disease, although data regarding SLN biopsy in PTs are lacking.

More than 20% of patients with malignant PT are likely to develop distant metastases with the most common locations being soft tissue, the lungs, pleura, bones, and abdominal viscera [1, 6]. The prognosis of malignant PT becomes poor when distant metastasis occurs [21], but the roles of chemotherapy and hormone therapy for metastatic malignant PT are not well defined. As most patients with distant metastasis have progressed from local recurrence [1, 9, 22], the surgeon should make every effort to achieve a negative margin to avoid local recurrence. Nevertheless, the occurrence of distant metastasis largely depends on the biological behavior of the tumor. Many histological prognostic factors, including stromal overgrowth, tumor necrosis, infiltrating margins, mixed mesenchymal components, high mitotic rate, and stromal atypia, have been evaluated, but in isolation, each factor appears to have low predictive value [1, 9]. Spanheimer et al. [9] reported that all distant recurrences developed in patients with malignant PT whose tumors had uniformly poor pathological features including marked stromal cellularity, stromal overgrowth, infiltrative borders, and ≧ 10 mitoses per 10 high-power fields. In their study, the presence of uniformly poor pathological features was found histologically in 29% of patients with malignant PT and predicted poor prognosis, with a 10-year disease-specific survival rate of 63% and a 10-year overall survival rate of 57%. Nevertheless, localized malignant PT has a relatively good prognosis. In a retrospective study, Grabowski et al. [23], reported that patients with localized malignant PT have a higher 10-year survival rate than those with invasive breast cancer with regional disease (90.9% vs. 61.5%, *p* < 0.001) [23].

Finally, we demonstrated that NSM with IBR is both curative and is an appealing cosmetic option for localized malignant PT, but long-term follow-up is required to determine the success of NSM and IBR.

## Data Availability

Data sharing is not applicable to this article as no datasets were generated or analyzed during the current study.

## Abbreviations

PT: Phyllodes tumor
NSM: Nipple sparing mastectomy
IBR: Immediate breast construction
BCS: Breast-conserving surgery
MRI: Magnetic resonance imaging
PET-CT: Positron emission tomography/ computed tomography
SLN: Sentinel lymph node
ALND: Axillary lymph-node dissection
